# Growth, physiology, and metabolism of *Halomonas meridiana* in aqueous ammonium sulfate with implications for icy moon astrobiology

**DOI:** 10.3389/fmicb.2025.1642998

**Published:** 2025-09-19

**Authors:** Cassie M. Hopton, Peter Nienow, Charles S. Cockell

**Affiliations:** ^1^UK Centre for Astrobiology, School of Physics and Astronomy, University of Edinburgh, Edinburgh, United Kingdom; ^2^School of Geosciences, University of Edinburgh, Edinburgh, United Kingdom

**Keywords:** ammonium, ammonium sulfate, icy moons, Europa, Titan, habitability, extremophiles, pollution

## Abstract

The discovery of extraterrestrial reservoirs of liquid water has motivated missions to icy moons Europa and Titan. Tentative evidence of ammonium sulfate ((NH_4_)_2_SO_4_) has been detected on the surface of Europa, and (NH_4_)_2_SO_4_ could be a prominent constituent of the Titan subsurface ocean. While ${\text{NH}}_{4}^{+}$ acts as a nitrogen source for many organisms, detrimental impacts of (NH_4_)_2_SO_4_ fertilizer have been documented in bacteria. Consequently, the presence of (NH_4_)_2_SO_4_ within icy moon environments may constrain the capacity of these environments to support life. In this study, the bacterial survival limits and physiological response to aqueous (NH_4_)_2_SO_4_ were assessed using the extremophile *Halomonas meridiana* Slthf1. Growth assays demonstrated concentrations exceeding 0.25 M (NH_4_)_2_SO_4_ led to a measurable slowing of the growth rate. Cell density remained comparable to control conditions up to 0.75 M (NH_4_)_2_SO_4_ at which a decline was observed. Contrary to existing hypotheses, alterations to cell density were not determined by pH, osmolarity, salinity, ionic strength, or water activity of the aqueous (NH_4_)_2_SO_4_ solution. Furthermore, neither ${\text{NH}}_{4}^{+}$ nor ${\text{SO}}_{4}^{2-}$ alone accounted for these alterations. Metabolite profiling revealed that exposure to (NH_4_)_2_SO_4_ reduced the abundance of glutamine compared to control, indicating an alteration to nitrogen, carbon, and energy metabolism. Active catabolism was suggested by reduced levels of purine metabolites and amino acids. Metabolites within the methylaspartate cycle were detected. We discuss these results with regards to the potential for habitability in aqueous extraterrestrial (NH_4_)_2_SO_4_ environments as well as terrestrial environments in which (NH_4_)_2_SO_4_ fertilizer is applied.

## Introduction

Where there is liquid water, there is the prospect for habitable conditions—the liquid water subsurface oceans of icy moons orbiting Jupiter (Europa, Ganymede, Callisto) and Saturn (Enceladus, Titan) are prominent targets in the search for life. Recently launched missions to Europa—the Jupiter Icy Moons Explorer (Juice) (Grasset et al., 2013) and Europa Clipper (Howell and Pappalardo, 2020), and the confirmed launch of NASA's Dragonfly mission to Titan (Barnes et al., 2021)—will probe these environments for extraterrestrial habitability. For decades, Europa and Titan have been hypothesized as environments that could support the emergence of life; there is availability of energy (Schulze-Makuch and Irwin, 2001; McKay and Smith, 2005; Hand et al., 2007; McKay, 2016) and many of the essential elements for life (CHNOPS: carbon, hydrogen, nitrogen, oxygen, phosphorus, sulfur) have been detected (Sagan et al., 1992; Hiscox, 2000; Owen, 2000; Nixon, 2024; Szalay et al., 2024).

A further compositional expectation for Europa and Titan is the presence of ammonia (Lewis, 1971; Engel et al., 1994; Spohn and Schubert, 2003; Tobie et al., 2005). Ammonia is a ubiquitous molecule found in a variety of celestial bodies (Wyckoff et al., 1989; Ao et al., 2011; Wong et al., 2018; Irwin et al., 2025). It can occur as the biologically toxic unionized ammonia (NH_3_) or less toxic ammonium ion (${\text{NH}}_{4}^{+}$). Under standard pressure and temperature, the speciation of ammonia (hereafter ammonia refers to the total ${\text{NH}}_{4}^{+}$ and NH_3_ in a system) is dependent on pH; a pH above or below 9.25 dictates whether NH_3_ (>pH 9.25) or ${\text{NH}}_{4}^{+}$ (< pH 9.25) predominates. In cold waters of 0 °C, this threshold increases to pH 10.1 (Bates and Pinching, 1949). While the ocean of Europa is predominantly magnesium sulfate (MgSO_4_) (McCord et al., 1998; Kargel et al., 2000; Zolotov and Shock, 2001), or possibly chloride salts (Brown and Hand, 2013; Hand and Carlson, 2015; Ligier et al., 2016), ammonium sulfate ((NH_4_)_2_SO_4_) could be a constituent at the surface of Europa (Mermy et al., 2023). Surface (NH_4_)_2_SO_4_ could be of oceanic origin due to emplacement by cryovolcanic venting (Roth et al., 2014; Sparks et al., 2016; Jia et al., 2018) or convection of the ice shell (Howell and Pappalardo, 2018). Indeed, with oceanic waters at pH < 8.4 (Johnson et al., 2019) and between −63 °C to 0 °C (Marion et al., 2003; Melosh et al., 2004), most ammonia within the internal ocean of Europa would be in the form of ${\text{NH}}_{4}^{+}$. On Titan, an ocean of aqueous (NH_4_)_2_SO_4_ fits with the modeled density and could account for cryovolcanism at the surface (Fortes et al., 2007; Grindrod et al., 2008). Titan's ocean temperature has been estimated in excess of −18°C (Sohl et al., 2014). The oceanic pH of Titan remains undetermined; an alkaline pH is predicted in models where NH_3_ is expected (~pH 11) (Marion et al., 2012; Leitner and Lunine, 2019). However, for the purpose of this study, we consider the aqueous (NH_4_)_2_SO_4_ ocean model.

The detection of ${\text{NH}}_{4}^{+}$ in the oceans of Europa and Titan would be a significant finding. ${\text{NH}}_{4}^{+}$ is one of the preferred nitrogen sources for many organisms on Earth (Kleiner, 1981; Raven et al., 1992; Britto et al., 2001; Reitzer, 2003; Li et al., 2013). Ammonia could have also acted as a nitrogen source for internal ocean prebiotic chemistry in early Earth (Martin et al., 2008; Sojo et al., 2016). The bioavailability of ${\text{NH}}_{4}^{+}$ on Earth underpins its widespread use as a nitrogen fertilizer, commonly in the form of ammonium nitrate (NH_4_NO_3_), diammonium phosphate ((NH_4_)_2_HPO_4_) or (NH_4_)_2_SO_4_ (Randive et al., 2021; Tyagi et al., 2022). However, there is a concentration limit at which ${\text{NH}}_{4}^{+}$ transitions from a vital nitrogen source to a cytotoxic compound. The toxicity of ${\text{NH}}_{4}^{+}$ in high concentrations has been well-documented in prokaryotes (Sprott and Patel, 1986; Hendriksen and Ahring, 1991; Leejeerajumnean et al., 2000), plants (Britto et al., 2001; Esteban et al., 2016; Hachiya et al., 2021) and aquatic eukaryotes (Ip et al., 2001; Randall and Tsui, 2002; Collos and Harrison, 2014).

In bacteria, the application of (NH_4_)_2_SO_4_ has shown to reduce populations, impact diversity (Gorissen et al., 1993; Witter et al., 1993; Toljander et al., 2008) and alter metabolism (Goude et al., 2004; Zorz et al., 2018). However, few studies have examined the survival limits of bacterial life in (NH_4_)_2_SO_4_. *Bacillus subtilis* and *Corynebacterium glutamicum* are capable of survival in up to, and possibly exceeding, 0.716 M (Leejeerajumnean et al., 2000) and 1 M (NH_4_)_2_SO_4_ (Müller et al., 2006), respectively. However, the oceans of Europa and Titan are putatively saline, cold and under hydrostatic pressure. It is therefore appropriate to assess habitability using terrestrial organisms with appropriate physiological adaptations. Halophilic bacteria have been shown to grow in brines relevant to the sodium chloride (NaCl), magnesium chloride (MgCl_2_) and MgSO_4_ content of Europa (Wilks et al., 2019; Cesur et al., 2022; Parker et al., 2023). Yet, the molar thresholds for survival and physiological impacts of (NH_4_)_2_SO_4_ on halophilic bacteria are poorly represented in the literature. Such information could allow us to assess the habitability of aqueous extraterrestrial environments and hypothesize suitable signatures that could be captured by life-detection machinery in (NH_4_)_2_SO_4_-bearing environments.

We have previously demonstrated that ammonia, predominantly speciated as NH_3_, can constrain growth and alter the physiology of *Halomonas meridiana* Slfth1 (Sltfh1) (nomenclature synonym: *H. aquamarina*). This had implications for the habitability of Enceladus and alkaline terrestrial environments (Hopton et al., 2025). These results could also be applicable to models of the Titan subsurface ocean where ammonia is NH_3_ (Lunine and Stevenson, 1987; Tobie et al., 2012; Sohl et al., 2014). Here, we aim to understand the survival limits and physiological response of Sltfh1 to (NH_4_)_2_SO_4_, with implications for the habitability of Europa and Titan that could bear solubilised oceanic (NH_4_)_2_SO_4_, as well as environments on Earth polluted with (NH_4_)_2_SO_4_ fertilizer. Sltfh1 is a deep-sea extremophile with physiological adaptations relevant to conditions presented within the oceans of Europa and Titan. We assessed cultivation of Sltfh1 in increasing concentrations of (NH_4_)_2_SO_4_ and other ammonium and sulfate salts. Using microscopy and an untargeted metabolomics approach, we determined physiological changes upon (NH_4_)_2_SO_4_ exposure. We draw conclusions on the habitability of extraterrestrial and terrestrial environments.

## Materials and methods

### Bacterial strain selection and cultivation

*Halomonas meridiana* Slthf1 (DSM 15724; Gram negative bacterium) was obtained from the German Collection of Microorganisms and Cell Cultures (DSMZ). It should be noted this strain has been synonymized with *H. aquamarina* based on phylogenomic classifications (Dobson and Franzmann, 1996), but *H. meridiana* remains validly published as a heterotypic synonym according to the International Code of Nomenclature of Prokaryotes (ICNP). Due to constantly evolving taxonomy, we refer to *H. meridiana* Slthf1 as “Slthf1” in the proceeding text. Slthf1 was isolated in a deep-sea hydrothermal environment. Such environments could have supported prebiotic chemistry on Earth (Martin et al., 2008; Sojo et al., 2016) and may occur within icy moon oceans (Vance et al., 2007; Hsu et al., 2015; Russell et al., 2017). Typical phenotypic characteristics of the hydrothermal-vent habitat of origin are exhibited by Slthf1 (Kaye and Baross, 2004; Kaye et al., 2004; Takahashi et al., 2020). This includes not only adaptability to high salinity (growth in up to 22% (w/v) NaCl) and high alkalinity (tolerance up to pH 12), but also, genomic adaptations to the cold (possessing three cold shock protein genes, growth at −1 °C) and high-pressure deep-sea environment (growth at 550 bar). This combination of polyextremophilic adaptations makes Slthf1 a superior model organism compared to halophilic archaea for studying potential life in cold, saline-alkaline environments similar to those presented in icy moon subsurface oceans. Additionally, Slthf1 has no known specialised adaptations to ammonium. This was an intentional choice. Ammonium content in the oceans of icy moons is such that adaptation to ammonium may not be required for survival. The intention of this study was to assess survival in ammonium, not to study an already established ammonium adaptation. The complete genome sequence for this organism is also available [DDBJ, accession no. AP022821] (Takahashi et al., 2020). Aerobic culture of Slthf1 was performed in glass conical Erlenmeyer flasks in an orbital benchtop shaking incubator set to rotate at 150 RPM, 28 °C. Slthf1 was cultivated in a yeast media consisting of 1 g/100 mL Bacto™ yeast extract (Becton, Dickinson and Company), 0.2 M NaCl (Thermo Fisher Scientific, CAS Number: 7647-14-5) and distilled water.

### Brine preparation

Solutions of ammonium and sulfate salts were prepared to 0.1 M, 0.25 M, 0.5 M, 0.75 M and 1 M from 2 M stock solutions diluted into yeast media. Ammonium salts included: (NH_4_)_2_SO_4_; Fisher Scientific, CAS Number: 7783-20-2, ammonium nitrate (NH_4_NO_3_; Scientific Laboratory Supplies, CAS Number: 6484-52-2) and ammonium chloride (NH_4_Cl; Honeywell Research Chemicals, CAS Number: 12125-02-9). In addition to (NH_4_)_2_SO_4_, sulfate salts included sodium sulfate (Na_2_SO_4_; Sigma Aldrich CAS number: 7757-82-6), and potassium sulfate (K_2_SO_4_; Acros Organics, CAS number: 7778-80-5). Owing to limited solubility of K_2_SO_4_, molarities beyond 0.5 M were not tested. (NH4)_2_SO_4_ was also prepared in yeast media at concentrations of 0.05 M, 0.125 M and 0.375 M to achieve equivalent ${\text{NH}}_{4}^{+}$ concentrations of 0.1 M, 0.25 M and 0.75 M, respectively, in accordance with the stoichiometry of ${\text{NH}}_{4}^{+}$ ions in other ammonium brines. The pH of solutions was determined with a Jenway 3510 benchtop pH meter. All solutions were between pH 5.5 and pH 6.5. Solution pH remained unmodified in order to preserve a high ${\text{NH}}_{4}^{+}$/NH_3_ ratio at acidic pH. Solutions of matching pH were created by addition of HCl or NaOH into yeast media. Solutions were matched to within ± 0.01 pH units. All solutions were filter-sterilized through a 0.22-micron pore before use.

### Growth conditions

The growth kinetics of Slthf1 cultivated in ammonium and sulfate salts was determined by recorded optical density (OD) measurements at 600 nm (OD_600_). Overnight Slthf1 culture was inoculated to OD_600_ = 0.05 into the selected brines. Controls were prepared by Slthf1 inoculation into unamended yeast media. Negative controls had no inoculation. Samples were seeded into a 96-well plate with a low evaporation lid and measurements taken with a BMG SPECTROstar Nano Microplate Reader over 48 h (h) at 28 °C. For cell viability assays, (NH_4_)_2_SO_4_ solutions were prepared to 1 M in yeast media. Brines were inoculated with overnight culture of Slthf1 to OD_600_ = 0.05 in a 96-well plate. Cultures were incubated in a tabletop shaker at 28 °C for 72 h. Cell viability was examined using colony forming units (CFU) on yeast media agar and incubated at 28 °C for 3 days prior to enumeration. To prevent condensation, 96-well plate lids were treated with a solution of Triton X-100 (0.05%) in 20% ethanol in all growth experiments.

### Growth kinetics

Growth curves were analyzed to determine growth rate and final cell density at 600 nm. Growth rate, μ, was calculated as per Equation 1, where *N*_0_ is the OD_600_ at the beginning of a selected time interval (*t*_0_) in the exponential growth phase; *N* is the OD_600_ at the end of a selected time interval (*t*) in the exponential growth phase. *t* and *t*_0_ were recorded in minutes.


(1) $$\begin{array}{l} \mu =\left(Log_{10}\left(N\right)-Log_{10}\left(N_{0}\right)\right)\;2.303/\left(t-t_{0}\right) \end{array}$$

Final cell concentration was indicated by the final OD_600_ reached after 48 h. Measurement of OD_600_ vs. cell viability confirms that an increase in OD_600_ reflects increased viability and proliferation of cells (Supplementary Figure S1).

### Water activity

Water activities were measured in the laboratory with a Rotronic HP23-AW water activity meter (Rotoronic AG, Bassersdorf, Switzerland). Solutions were prepared and measured after a time interval of 1.5 h to allow equilibration.

### Metabolomics sampling and extraction

Slthf1 was cultivated overnight and inoculated to OD_600_ = 0.05 into 0.5 M (NH_4_)_2_SO_4_ or unamended yeast media (control) within a 24-well plate. Growth at 28 °C was assessed by OD_600_ readings every 30 min using a BMG SPECTROstar Nano Microplate Reader. Slfth1 was harvested at OD_600_ = 0.5 following 28 h growth. An aliquot of each sample was placed into a microcentrifuge tube and briefly incubated on ice. Samples were retained for transmission electron microscopy (TEM) as described in the TEM preparation section. The remaining samples were quenched by rapid cooling in a dry ice-ethanol bath (70% v/v ethanol). Samples were vigorously mixed to prevent freezing. Any spent medium was discarded by centrifugation at 1,000 × *g* for 10 min at 4 °C followed by removal of supernatant. Metabolites were extracted by application of ice-cold chloroform/methanol/water (1:3:1). During metabolite extraction, cell lysis was encouraged by sonication of samples in water for 5 min at 37 kHz in an ultrasonication bath (Elmasonic S 60 H) maintained at 4 °C with ice. Extraction mixtures were shaken at 1,200 RPM for 1 h at 4 °C and centrifuged at 13,000 × *g* for 3 min at 4 °C. The metabolite-rich supernatant was harvested into sterile microcentrifuge tubes and maintained at −80 °C until analysis. A quality control sample was created by pooling equal volumes of metabolites from all samples, which was also maintained at −80 °C until analysis.

### Metabolomics

Global metabolomic profiling was conducted using liquid chromatography (LC) coupled with ion mobility (IM) quadrupole time-of-flight (qTOF) mass spectrometry (MS). The system consisted of an Agilent 1290 Infinity II series ultra-high-performance liquid chromatography (UHPLC) setup interfaced with an Agilent 6560 IM-qTOF mass spectrometer equipped with a Dual Agilent Jet Stream Electron Ionization source. Chromatographic separation was achieved using an InfinityLab Poroshell 120 HILIC-Z UHPLC column (2.1 mm × 50 mm, 2.7 μm) coupled to an InfinityLab Poroshell 120 HILIC-Z guard column (3.0 mm × 2.7 μm), both sourced from Agilent Technologies (689775–924 and 823750–948, respectively). A gradient elution was performed over 3.5 min, utilizing an organic solvent (acetonitrile) in combination with an aqueous buffer, either low-pH (10 mM ammonium formate, pH 3) for positive ionization or high-pH (10 mM ammonium acetate, pH 9) for negative ionization. Data were collected using MassHunter Data Acquisition 10.0 software, with 1 μL of each sample injected at a flow rate of 800 μL/min. A pooled quality control (QC) sample, comprising equal volumes of all experimental samples, was injected five times at the beginning of the experiment to equilibrate the column and after every subsequent set of five test samples to monitor system stability during data acquisition. Mass spectrometry data were acquired over a m/z range of 50–1,700, with a scan rate of 0.8 scans per second. The metabolomic analysis was performed at the EdinOmics research facility (RRID: SCR_021838) at the University of Edinburgh.

### Data processing and statistical analysis of the metabolomics dataset

Analysis of the raw data files was performed by the Agilent MassHunter software suite. Specifically, ion multiplexed and calibration files underwent demultiplexing with the PNNL PreProcessor v2020.03.23, utilizing default settings for tasks such as demultiplexing, moving average smoothing, saturation correction, and spike removal. For recalibration, accurate mass and drift time adjustments were made using AgtTofReprocessUi and IM-MS Browser 10.0, respectively. Molecular features were extracted using Mass Profiler 10.0, with parameters set for retention time tolerance (±0.3 min), drift time tolerance (±1.5%), and accurate mass tolerance (± 5 ppm + 2 mDa). Feature annotation was carried out by matching accurate mass and collision cross-section (CCS) values to the McLean CCS Compendium PCDL library (Nichols et al., 2018). Statistical analyses was performed via the MetaboAnalyst 6.0 online platform (Pang et al., 2024), with data log-transformed and Pareto-scaled before analysis. Annotated molecular features were used to generate principal component analysis (PCA), volcano analysis, unpaired *t*-test and box plots. For pathway analysis, compound names were first converted to ID labels according to the human metabolome database (HMDB). Compound HMDB ID with relative intensities were submitted to the MetaboAnalyst 6.0 online platform pathway analysis tool. Data was log-transformed, auto-scaled and examined against the *H. meridiana* SCSIO 43005 KEGG pathway library using global test and relative betweenness centrality methods. Altered pathways with a *p*-value < 0.05 and FDR < 0.05 were considered significant. Significantly altered metabolites in the unpaired *t*-test that were also identified as altered in the pathway analysis are depicted in box and whisker plots. The plots were retrieved following *t*-test analysis on the MetaboAnalyst 6.0 online platform. Normalized values are presented. The raw data associated with this study is available in the Supplementary Data Sheet. This study focuses on metabolomic changes in (NH_4_)_2_SO_4_, but the broader metabolomic profiling also included samples cultivated in NH_3_ and NaOH. For the purpose of this study, only (NH_4_)_2_SO_4_ and control samples were included. The metabolomics of NH_3_ and NaOH exposed samples were addressed in a separate analysis (Hopton et al., 2025).

### Transmission electron microscopy

Cultures of Slthf1 cultivated in 0.5 M (NH_4_)_2_SO_4_ were harvested during metabolomics sampling, prior to extraction. Cells were pelleted by centrifugation at 5,000 × *g* and supernatant removed. The pellet was washed and resuspended in phosphate-buffered saline (PBS). Following centrifugation at 5,000 × *g* and supernatant removal, the cell pellets were fixed in 3% glutaraldehyde prepared in 0.1 M sodium cacodylate buffer (pH 7.3) for 2 h, followed by three 10 min washes in 0.1 M sodium cacodylate. Post-fixation was carried out using 1% osmium tetroxide in 0.1 M sodium cacodylate for 45 min, followed by a series of three 10 min washes in 0.1 M sodium cacodylate. The samples were dehydrated sequentially in ethanol solutions at 50%, 70%, 90%, and 100% for 15 min each. This was repeated in triplicate and followed by two 10 min washes in propylene oxide. The samples were embedded in TAAB 812 resin. Sections of 1 μm thickness were prepared using a Leica Ultracut ultramicrotome, stained with Toluidine Blue, and examined under a light microscope to identify regions of interest. Ultrathin sections (60 nm thick) were cut from these selected regions, stained with uranyl acetate and lead citrate, and observed using a JEOL JEM-1400 Plus TEM. Representative images were acquired with a GATAN OneView camera at 4K resolution and subsequently processed using ImageJ software (version 57).

### Statistics and reproducibility

Normality of data was assessed with the Shapiro-Wilk test. For comparison of two groups, equal variance was assessed with an F-test. Groups of equal variances were analyzed by unpaired two-tailed *t*-test. Groups of unequal variances were assessed by unpaired two-tailed *t*-test with Welch's correction. For analysis of three or more groups, equal variance was assessed by the Brown-Forsythe test. Samples of equal variance were analyzed by analysis of variance (ANOVA) followed by Tukey's *post-hoc* test. For samples where variance was not equal, Welch's ANOVA test with Tamhane's T2 *post-hoc* test was applied. For datasets with non-normal distribution, means were compared using the Kruskal–Wallis test with Dunn's multiple comparisons test. Statistical tests are specified in figure legends. Results where *p* < 0.05 were considered significant. All data was compiled from at least three biological replicates (*n* = 3–5). Data is presented as the mean ± standard deviation (SD). All figures and statistical analyses were produced using GraphPad Prism version 8.0.2 (GraphPad Software Inc.).

## Results

### Concentration thresholds for growth of Slthf1 in (NH_4_)_2_SO_4_

**Figure 1:**
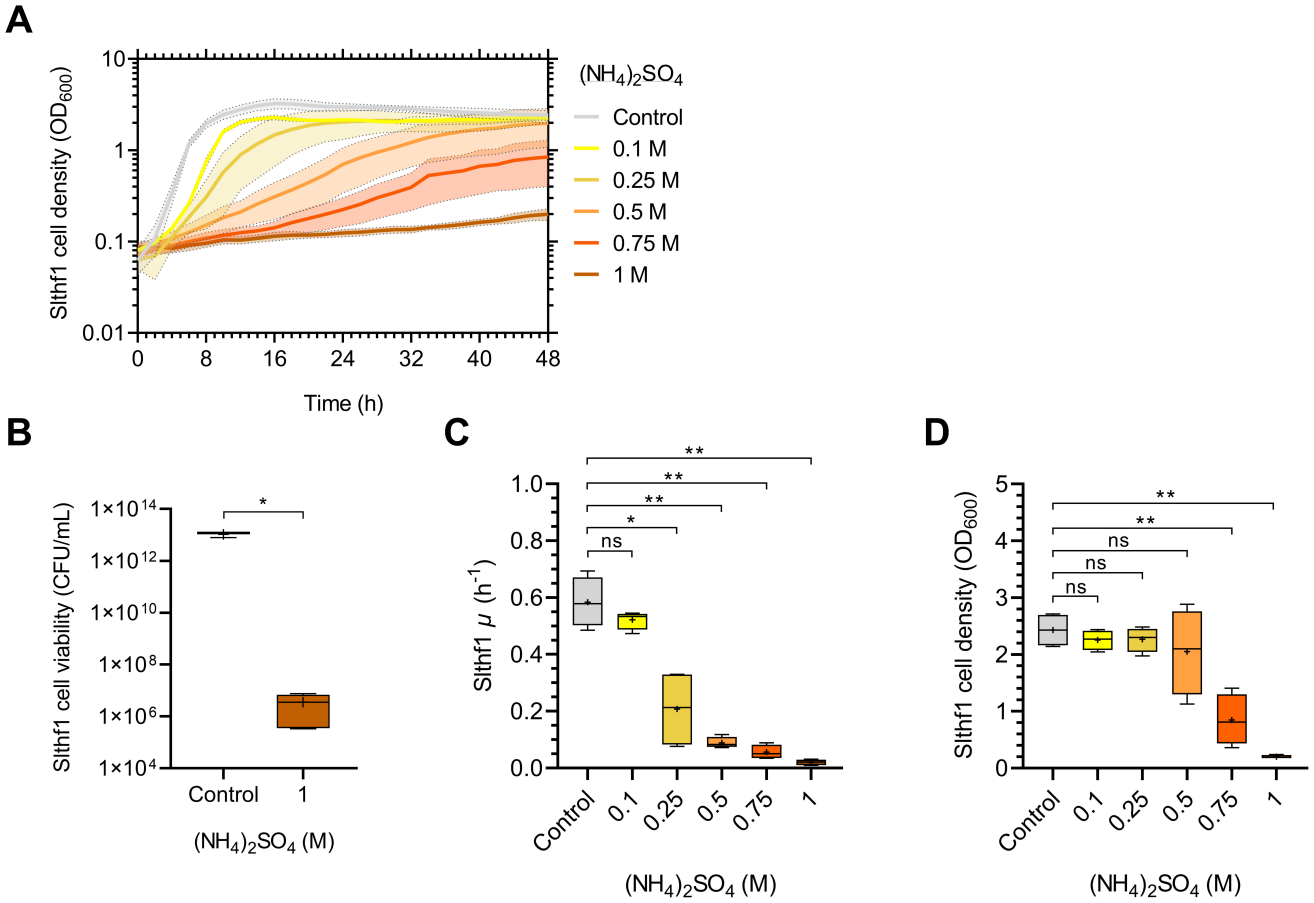
Growth dynamics of Slfth1 in increasing molar concentrations of (NH_4_)_2_SO_4_. **(A)** OD_600_ growth curve of Slthf1 cultivated in 0 M (control), 0.1 M, 0.25 M, 0.5 M, 0.75 M, and 1 M (NH_4_)_2_SO_4_ over 48 h. Growth curves represent mean OD_600_ values over time ± s.d. (*n* = 4). Error is indicated by area fill within error bands. **(B)** CFU of Slfth1 following 72 h cultivation in 0 M (NH_4_)_2_SO_4_ (control, *n* = 3) and 1 M (NH_4_)_2_SO_4_ (*n* = 5). **(C)** Growth rate (μ) and **(D)** final OD_600_ at 48 h extrapolated from **(A)** in increasing molar concentrations of (NH_4_)_2_SO_4_. Statistics in **(B)** correspond to a two-tailed unpaired *t*-test with Welch's correction. Statistics in **(C)** and **(D)** correspond to Welch's ANOVA using Tamhane's T2 multiple comparisons test. ns, no significance; *p < 0.05; **p < 0.01.

Growth of Slthf1 over 48 h in increasing concentrations of (NH_4_)_2_SO_4_ was investigated to assess concentration thresholds of growth in (NH_4_)_2_SO_4._ Concentrations of 0.1 M, 0.25 M, 0.5 M, 0.75 M and 1 M were utilized, with unamended yeast media, 0 M (NH_4_)_2_SO_4_, as a control. The resulting growth curves are depicted in Figure 1A. Growth progressively declined with increasing (NH_4_)_2_SO_4_, with minimal cell density observed at 1 M (NH_4_)_2_SO_4._ Cell viability assay confirmed that Slthf1 remained viable at 1 M (NH_4_)_2_SO_4_ after 72 h incubation (Figure 1B). Growth rate and final cell density at 48 h are shown in Figures 1C, D, respectively. Overall, growth of Slthf1 was limited by increasing concentrations of (NH_4_)_2_SO_4._ Growth rate was non-significant from control at concentrations of 0.1 M (*p* = 0.599). Successive reduction in growth rate compared to control was observed in 0.25 M (*p* < 0.05), 0.5 M (*p* < 0.01), 0.75 M (*p* < 0.01) and 1 M (*p* < 0.01) brines. However, reduction in growth rate does not affect final cell density when grown up to 0.5 M (NH_4_)_2_SO_4_; at 48 h, there was no significant difference between the OD_600_ in control solutions compared to 0.1 M (*p* = 0.766), 0.25 M (*p* = 0.825) and 0.5 M (*p* = 0.815). Cell density was lower compared to control in 0.75 M (*p* < 0.01) and 1 M (NH_4_)_2_SO_4_ (*p* < 0.01). Cell density remained above OD_600_ = 2.00 when cultivated in the control, 0.1 M, 0.25 M and 0.5 M (NH_4_)_2_SO_4_. Cell density was below OD_600_ = 1.00 in 0.75 M (NH_4_)_2_SO_4_ (OD_600_ = 0.847 ± 0.448). The average cell density of Slthf1 after 48 h incubation in 1 M (NH_4_)_2_SO_4_ was 0.2 OD_600_ ± 0.029.

### Comparative growth and water activity analysis in ammonium and sulfate salts

**Figure 2:**
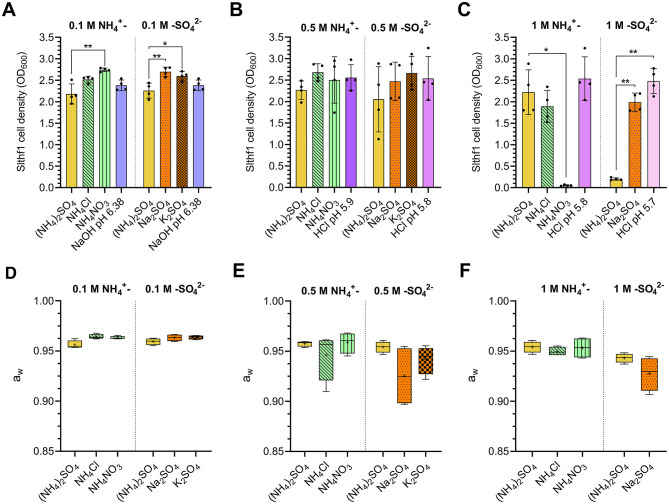
Cell density and water activity of Slfth1 cultivated in ammonium and sulfate salts of matched molar concentrations of ${\text{NH}}_{4}^{+}$ and ${\text{SO}}_{4}^{2-}$. **(A–F)** Aqueous solutions are represented by the following patterns: (NH_4_)_2_SO_4_–solid yellow; NH_4_Cl—green with diagonal stripes; NH_4_NO_3_–green with vertical stripes; Na_2_SO_4_–dotted orange; K_2_SO_4_–checkered orange; pH—solid purple to pink scaling with alkaline to acidic pH. **(A–C)** Final OD_600_ following 48 h incubation of Slfth1 in salts with matched molar concentrations of ${\text{NH}}_{4}^{+}$ and ${\text{SO}}_{4}^{2-}$ ions at concentrations of **(A)** 0.1 M, **(B)** 0.5 M, and **(C)** 1 M, as well as solutions of matched pH made by NaOH and HCl. Statistical tests employed: one-way ANOVA with Tukey's *post-hoc* test for 0.1 M (NH_4_)_2_SO_4_ vs. 0.1 M sulfate salts **(A)** and 0.25 M (NH_4_)_2_SO_4_ vs. 0.5 M ammonium salts **(B)**; the Kruskal–Wallis test using Dunn's multiple comparisons test for 0.05 M (NH_4_)_2_SO_4_ vs. 0.1 M ammonium salts **(A)** and 0.5 M (NH_4_)_2_SO_4_ vs. 0.5 M sulfate salts **(B)**; iii) all comparisons in **(C)** were made by Welch's ANOVA using Tamhane's T2 multiple comparisons test. **(D–F)** Water activities of salts with matched molar concentrations of ${\text{NH}}_{4}^{+}$ and ${\text{SO}}_{4}^{2-}$ ions at **(D)** 0.1 M, **(E)** 0.5 M, and **(F)** 1 M. Statistical comparison of ammonium and sulfate salts occurred separately. Statistical tests employed: one-way ANOVA with Tukey's *post-hoc* test for 0.1 M (NH_4_)_2_SO_4_ vs. 0.1 M sulfate salts **(D)**; Kruskal–Wallis test using Dunn's multiple comparisons test for 0.05 M (NH_4_)_2_SO_4_ vs. 0.1 M ammonium salts **(D)** and 0.25 M (NH_4_)_2_SO_4_ vs. 0.5 M ammonium salts **(E)**; Welch's ANOVA using Tamhane's T2 multiple comparisons test for 0.5 M (NH_4_)_2_SO_4_ vs. 0.5 M sulfate salts **(E)** and 0.5 M (NH_4_)_2_SO_4_ vs. 1 M ammonium salts **(F)**; unpaired two-tailed *t*-test for comparison of 1 M (NH_4_)_2_SO_4_ vs. 1 M Na_2_SO_4_ **(F)**. All a_w_ comparisons were found to be non-significant. ns, no significance; *p < 0.05; **p < 0.01.

The established survival limits of Slthf1 in increasing concentrations of (NH_4_)_2_SO_4_ could be due to altered water availability, salinity, osmotic pressure, ion induced toxicity or pH changes. To investigate these possibilities, Slthf1 was cultivated in ammonium (NH_4_Cl, NH_4_NO_3_) and sulfate salts (Na_2_SO_4_, K_2_SO_4_) at concentrations of 0.1 M (Figure 2A), 0.5 M (Figure 2B) and 1 M (Figure 2C), in addition to (NH_4_)_2_SO_4_. Growth was assessed by OD_600_ after 48 h incubation. For comparison against ammonium salts, (NH_4_)_2_SO_4_ was prepared to concentrations of 0.05 M, 0.25 M and 0.5 M to ensure ionic levels of ${\text{NH}}_{4}^{+}$ were equivalent to NH_4_Cl and NH_4_NO_3_ at 0.1 M, 0.5 M, 1 M, respectively. Salinity, osmolarity and ionic strength of each brine is displayed in Table 1. Slthf1 was also grown in unamended yeast media pH-matched to (NH_4_)_2_SO_4_ brines using NaOH or HCl. Slthf1 reached an OD_600_ > 2 in all brines at 0.1 M (Figure 2A). The OD_600_ at 48 h of Slthf1 in 0.05 M (NH_4_)_2_SO_4_ (0.1 M ${\text{NH}}_{4}^{+}$) was found to be not significantly different in 0.1 M NH_4_Cl (*p* = 0.380) and the pH-matched solution at pH 6.38 (*p* > 0.999) (Figure 2A). There was a higher OD_600_ in 0.1 M NH_4_NO_3_ compared to 0.05 M (NH_4_)_2_SO_4_ (*p* < 0.01). When matching the molar concentration of ${\text{SO}}_{4}^{2-}$ ion, the OD_600_ at 48 h was lower in 0.1 M (NH_4_)_2_SO_4_ compared to 0.1 M Na_2_SO_4_ (*p* < 0.01) and 0.1 M K_2_SO_4_ (*p* < 0.05). Growth in the pH-matched solution at pH 6.38 was not significantly different from growth in 0.1 M (NH_4_)_2_SO_4_. At 0.5 M, there was no significant difference between the OD_600_ at 48 h for any of the tested brines compared to growth in 0.25 M (0.5 M ${\text{NH}}_{4}^{+}$) and 0.5 M (NH_4_)_2_SO_4_ (Figure 2B) (*p*-values in Supplementary Table S1), despite differential salinity, osmolarity and ionic strengths between certain brines.

**Table 1:** Osmolarity, salinity and ionic strength of 0.05 M (NH_4_)_2_SO_4_ and 0.1 M (NH_4_)_2_SO_4_, NH_4_Cl, NH_4_NO_3_, Na_2_SO_4_, and K_2_SO_4_ solutions utilized in this study.

**Solution**	**Osmolarity (Osm/L)**	**Salinity (ppt)**	**Ionic strength (M)**
0.1 M NH_4_Cl	0.20	0.0054	0.10
0.1 M NH_4_NO_3_	0.20	0.0080	0.10
0.1 M (NH_4_)_2_SO_4_	0.30	0.0132	0.30
0.05 M (NH_4_)_2_SO_4_	0.15	0.0066	0.15
0.1 M Na_2_SO_4_	0.30	0.0142	0.30
0.1 M K_2_SO_4_	0.30	0.0174	0.30

Alterations to cell density became evident when brine concentrations reached 1 M (Figure 2C). Slthf1 grew in 1 M NH_4_Cl (*p* = 0.883) and a pH-matched solution at pH 5.8 (*p* = 0.933) with a non-significant change to OD_600_ at 48 h compared to 0.5 M (NH_4_)_2_SO_4_ (1 M ${\text{NH}}_{4}^{+}$). Cell density was maintained above OD_600_ = 1.8 in these solutions. OD_600_ in 1 M NH_4_NO_3_ was lower compared to (NH_4_)_2_SO_4_ (*p* < 0.05), with a final OD_600_ < 0.05 indicating severely limited growth. Thus, the molarity of ${\text{NH}}_{4}^{+}$ ion alone does not determine growth outcomes. Growth in 1 M (NH_4_)_2_SO_4_ was significantly lowered compared to growth in 1 M Na_2_SO_4_ (*p* < 0.01) and a pH-matched solution at pH 5.7 (*p* < 0.01), despite the fact that Na_2_SO_4_ displayed higher salinity, and equal osmolarity and ionic strength compared to (NH_4_)_2_SO_4_ (Table 1). This confirms molarity of ${\text{SO}}_{4}^{2-}$ alone does not determine growth outcomes. The difference between the OD_600_ at 48 h in 1 M Na_2_SO_4_ and the pH-matched solution at 5.7 was found to be non-significant (*p* = 0.108). Water availability was assessed by water activity measurements of the brines at each concentration −0.1 M (Figure 2D), 0.5 M (Figure 2E) and 1 M (Figure 2F). The water activity of all brines was found to be above 0.9 a_w_ (Figures 2D–F). There was a non-significant difference between the a_w_ of ammonium salts and the a_w_ of sulfate salts (*p*-values in Supplementary Table S2). The results of these tests suggest that neither toxicity by individual ions, osmotic stress, ionic strength, salinity nor pH were contributing factors that limit growth at higher concentrations of (NH_4_)_2_SO_4_.

### Altered metabolites of Slthf1 cultivated in (NH_4_)_2_SO_4_

**Figure 3:**
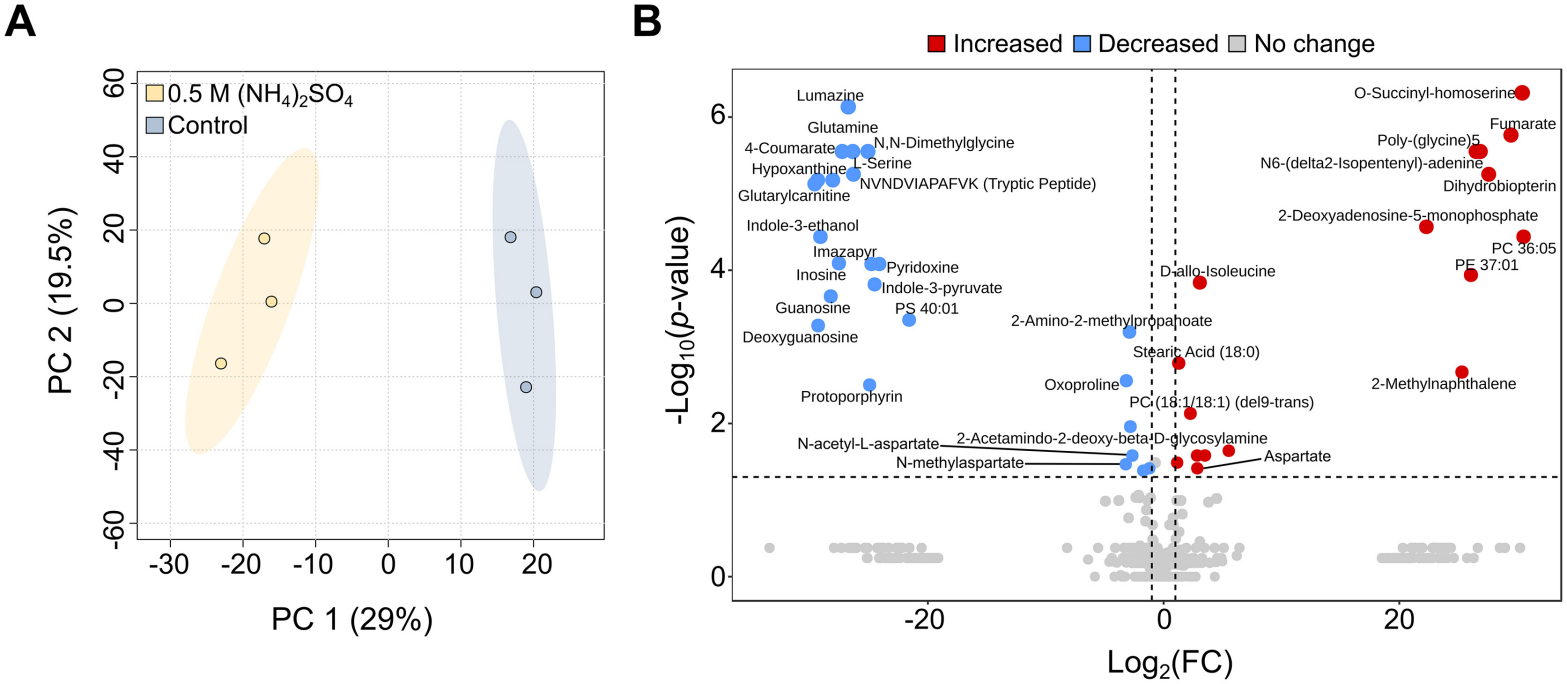
Metabolite changes of Slfth1 cultivated in 0.5 M (NH_4_)_2_SO_4_. **(A)** Principal component analysis (PCA) scores plot depicting clear separation of the control (0 M (NH_4_)_2_SO_4_) from 0.5 M (NH_4_)_2_SO_4_ cultivated samples. **(B)** Volcano plot depicting metabolites with a fold change >2 and a *p*-value lower than 0.05 (adjusted using FDR correction) for 0.5 M (NH_4_)_2_SO_4_/control. Comparatively elevated metabolites (red) and reduced metabolites (blue) are depicted. Metabolites without significant change are shown in gray.

Metabolites can indicate stress (Avci, 2024; Sharma et al., 2025), and can additionally be utilized as biomarkers in the search for life (Fairén et al., 2020; Weber et al., 2023). To examine the stress response and adaptations in (NH_4_)_2_SO_4_, comparative untargeted metabolomics was performed in Slthf1 cultivated under two conditions: 0.5 M (NH_4_)_2_SO_4_ and unamended yeast media (0 M (NH_4_)_2_SO_4_, hereafter denoted “control”). The annotated molecular features with relative intensities underwent multivariate and univariate statistical analysis, the results of which are shown in a PCA scores plot (Figure 3A), volcano plot (Figure 3B), and unpaired *t*-test (Supplementary Table S3). The PCA scores plot shows clear separation of the 0.5 M (NH_4_)_2_SO_4_ group from control group with no overlap. The metabolites attributed to this group differentiation were identified using univariate volcano analysis, using a fold change greater than 2 and a *p*-value < 0.05 (adjusted using FDR correction). Volcano analysis revealed significant elevation of 17 molecular features (*p* < 0.05, FDR corrected), and significant reduction of 24 molecular features (*p* < 0.05, FDR corrected) in 0.5 M (NH_4_)_2_SO_4_ cultivated samples compared to control samples. The complete volcano analysis dataset for this comparison is shown in Supplementary Table S4. The altered metabolites included amino acids and derivatives; there was an enrichment of aspartate (FC = 7.24, *p* < 0.05) and D-allo-isoleucine (FC = 8.42, *p* < 0.0001), and a reduction to the levels of serine (FC = 1.2 × 10^−8^, *p* < 0.0001), glutamine (FC=1.15 × 10^−8^, *p* < 0.0001) and N-acetyl-L-aspartate (FC = 0.159, *p* < 0.05) in 0.5 M (NH_4_)_2_SO_4_ cultivated samples compared to control samples.

### Morphological changes in (NH_4_)_2_SO_4_ cultivated Slfth1

**Figure 4:**
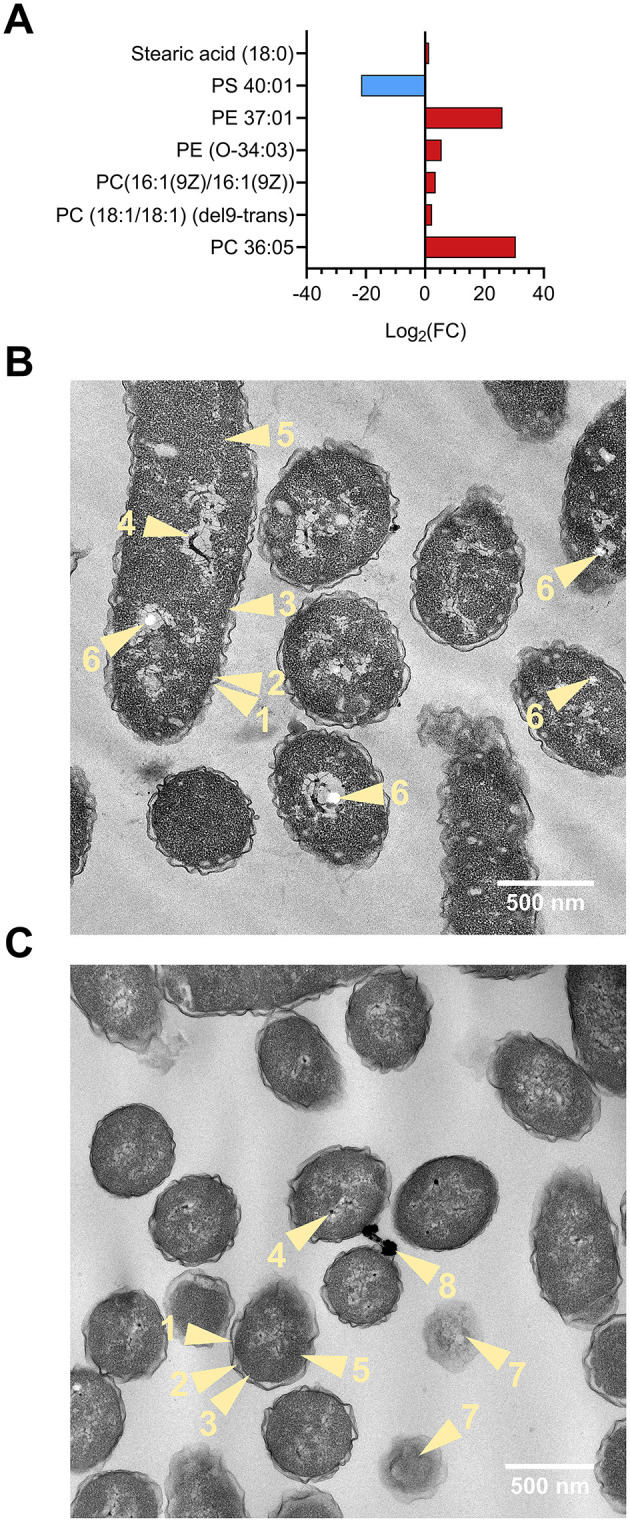
Lipid analysis and morphology of Slthf1 growth in (NH_4_)_2_SO_4_. **(A)** Phospholipid alterations of Sltfh1 cultivated in 0.5 M (NH_4_)_2_SO_4_. Alterations are depicted as Log_2_ fold-change (FC) from control samples (0 M (NH_4_)_2_SO_4_). Lipids depicted had a FC greater than 2 and were also identified as significantly altered (*p*-value < 0.05) in the volcano analysis. PC, phosphatidylcholine; PE, phosphatidylethanolamine; PS, phosphatidylserine. **(B, C)** Transmission electron microscopy (TEM) micrographs depicting morphology of Slfth1 harvested for metabolomics when OD_600_ = 0.5 at 28 h in **(B)** 0 M (NH_4_)_2_SO_4_ (control) **(C)** 0.5 M (NH_4_)_2_SO_4_. Light yellow numbered items and arrows refer to the following biological components: 1, undulating outer membrane; 2, periplasmic space; 3, inner membrane; 4, nucleoid; 5, cytoplasm; 6, PHA-like granule; 7, lysed cell; 8, electron-dense material.

Lipids were also found to be significantly altered in the volcano analysis. Figure 4A depicts lipid alterations as Log2(FC) from control sample. The levels of unsaturated phosphatidylcholine (PC) 36:05 (FC = 1.6 × 10^9^, *p* < 0.0001), PC (18:1/18:1) (del9-trans) (FC = 4.83, *p* < 0.01), PC [16:1(9Z)/16:1(9Z)] (FC = 11.35, *p* < 0.05) and phosphatidylethanolamine (PE) 37:01 (FC = 3.16 × 10^7^, *p* < 0.001) and PE (O-34:03) (FC = 46.15, *p* < 0.05) were higher in (NH_4_)_2_SO_4_ cultivated Slthf1. There were lower levels of unsaturated 40-carbon phosphatidylserine (FC = 3.16 × 10^−7^, *p* < 0.001), and a small but significant elevation in the levels of saturated stearic acid (FC = 2.43, *p* < 0.01) in Slthf1 cultivated in (NH_4_)_2_SO_4_ compared to the control. These alterations suggest cell wall modulation; morphological changes in Slthf1 cultivated in control and 0.5 M (NH_4_)_2_SO_4_ solutions are shown in Figures 4B, C, respectively. Cells in both conditions exhibited irregular, undulating outer membrane morphology with an enlarged periplasm between inner and outer membrane. Cytoplasm showed an abundance of ribosomes and nucleoids in both conditions. PHA-like granules were also apparent in both conditions but significantly greater in number in the control condition. This possibly suggested nitrogen limitation in the growth media that was satisfied by addition of ${\text{NH}}_{4}^{+}$ in cells cultivated in 0.5 M (NH_4_)_2_SO_4_. Cells without membranes, indicating cell lysis events, were evident with greater occurrence in the (NH_4_)_2_SO_4_ cultivated cells. There was electron-dense material observed between cells cultivated in (NH_4_)_2_SO_4_ that may indicate microbial interactions with (NH_4_)_2_SO_4._

### (NH_4_)_2_SO_4_ cultivation reduces abundance of the nitrogen metabolism metabolite glutamine

**Figure 5:**
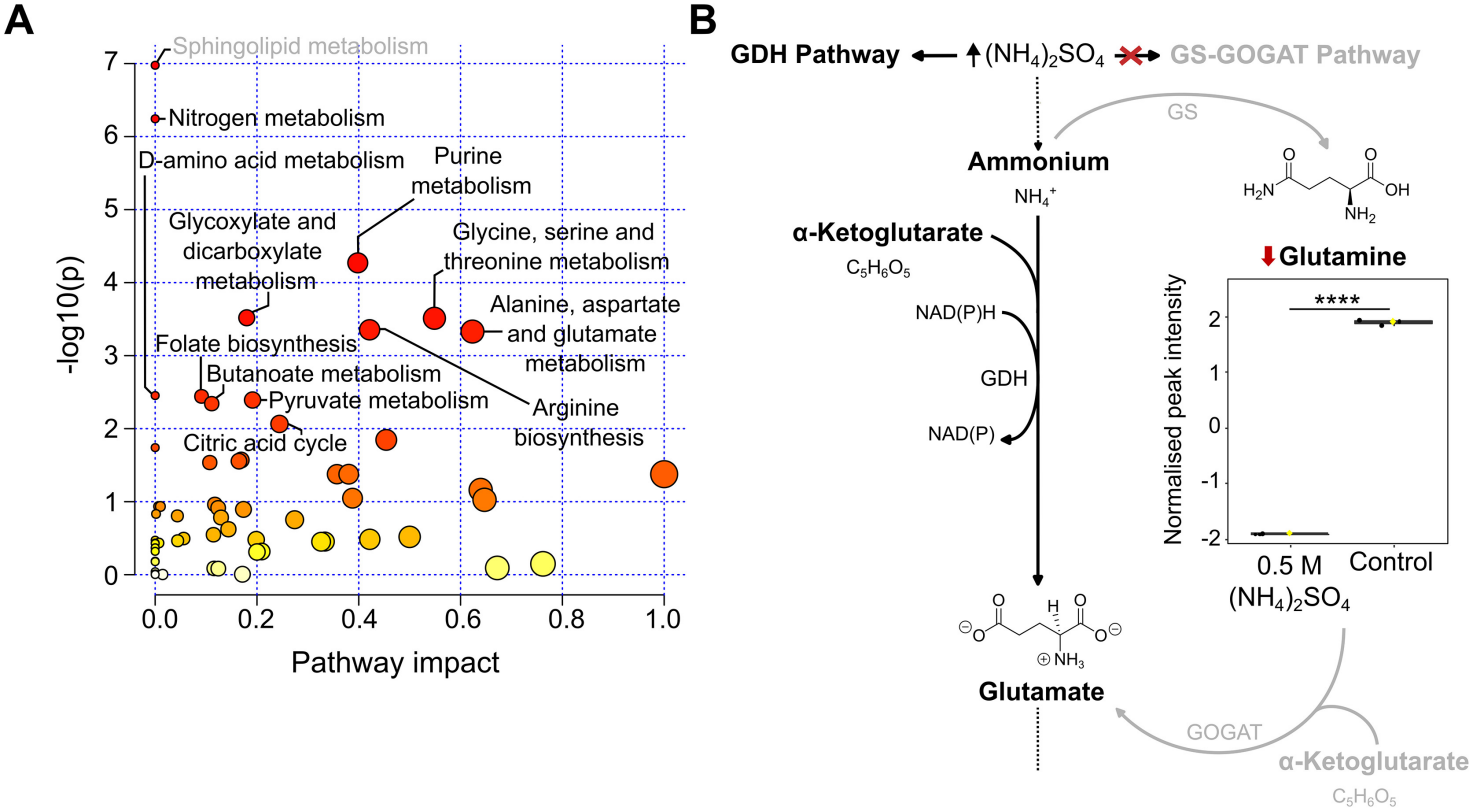
Altered pathways in Slfth1 cultivated in 0.5 M (NH_4_)_2_SO_4_. **(A)** Scatter plot of KEGG pathways identified in the metabolomics dataset. Altered pathways with a *p*-value < 0.05 and FDR < 0.05 were considered significant. The size of each node is relative to the pathway impact values (i.e., the importance of the identified metabolite to the depicted pathway), while the color of nodes is indicative of *p*-value, with a darker red coloring indicating a more significant change to the pathway indicated. **(B)** Proposed altered nitrogen metabolism in (NH_4_)_2_SO_4_ cultivated Slfth1. The box and whiskers summarize the normalized values with mean indicated by a yellow diamond and the central line indicating the median black dots representing the values from all samples (*n* = 3). Box and whiskers were generated using MetaboAnalyst 6.0 and edited for visual clarity in Inkscape. Diagram depicts high concentrations of (NH_4_)_2_SO_4_ altering ammonium assimilation by having an inhibitory effect on the glutamine synthetase (GS)-glutamate synthase (GOGAT) pathway, thus reducing glutamine relative to control samples. Nitrogen assimilation occurs preferentially by the glutamate dehydrogenase (GDH) pathway. Chemical structures were created using ChemDraw. ****p < 0.0001.

To identify pathways altered upon 0.5 M (NH_4_)_2_SO_4_ exposure, a pathway enrichment analysis of the annotated metabolites was conducted using the MetaboAnalyst 6.0 platform. Significantly altered metabolites in the unpaired *t*-test (Supplementary Table S3) that were also identified as altered in the pathway analysis are depicted in box and whisker plots in the following sections. Pathway analysis identified 12 significantly (*p* < 0.05) altered pathways. Figure 5A depicts the altered pathways. Associated significance values are in Supplementary Table S5. The altered pathways correspond to sphingolipid, nitrogen, purine, glyoxylate and dicarboxylic, amino acid, folate, pyruvate, butanoate metabolism and the citric acid cycle. The levels of serine were found to be lower in 0.5 M (NH_4_)_2_SO_4_ cultivated cells relative to control samples (*p* < 0.0001). This resulted in the pathway of sphingolipid metabolism to appear significantly altered. However, based on the complete genome of Slthf1, we do not believe this organism to be capable of sphingolipid metabolism. The next most significantly altered pathway corresponded to nitrogen metabolism, attributed to the significant reduction to glutamine (*p* < 0.0001) in (NH_4_)_2_SO_4_ samples compared to control samples. Glutamate was not identified as significantly altered. The reduced levels of glutamine could suggest, in ${\text{NH}}_{4}^{+}$ surplus, nitrogen assimilation shifted from utilizing glutamine. The proposed alternative pathway for nitrogen metabolism is presented in Figure 5B.

### (NH_4_)_2_SO_4_ lowers purine levels in Slthf1

**Figure 6:**
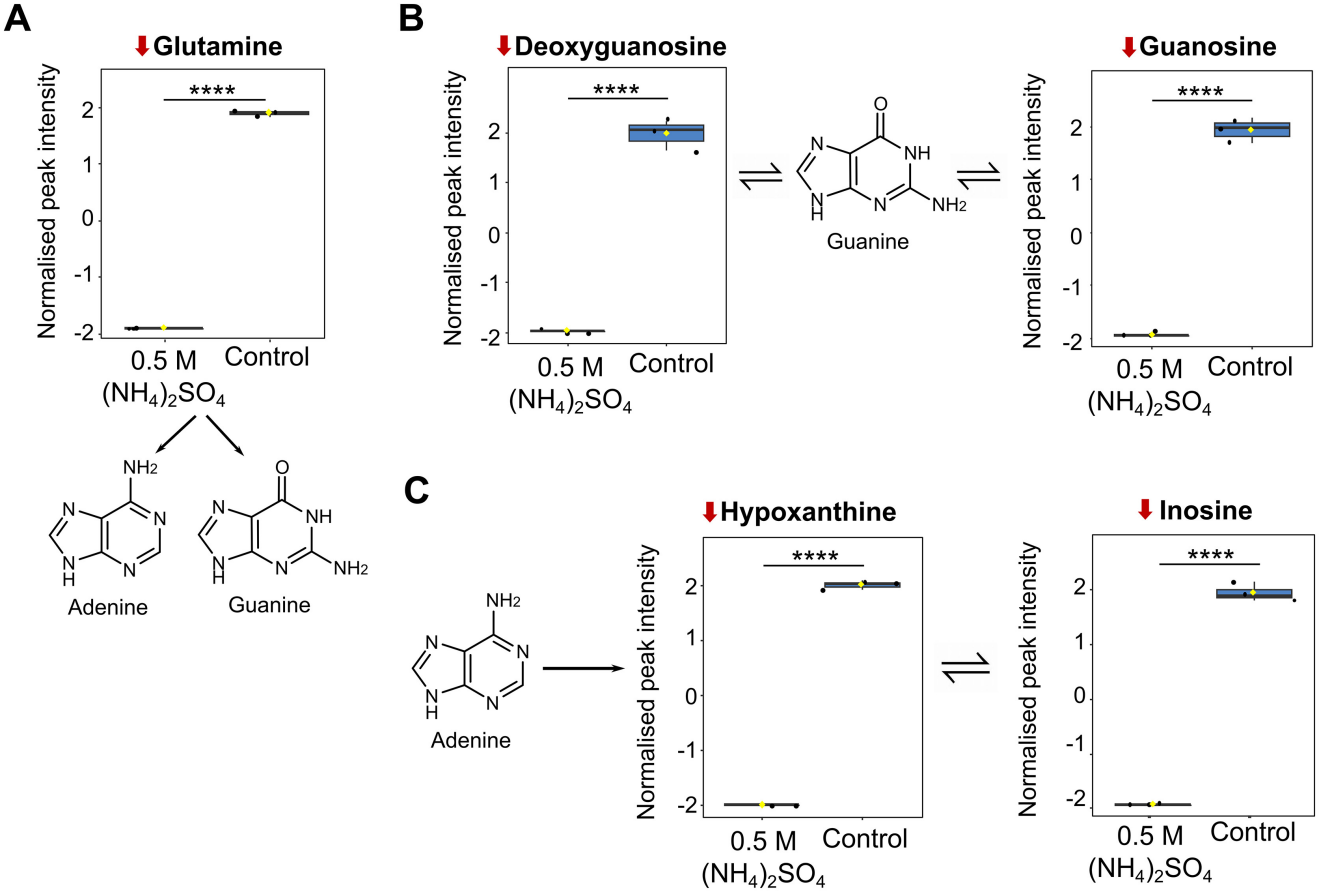
Metabolite changes associated with purine metabolism. Box plots showing significantly altered metabolites involved in **(A)** purine biosynthesis **(B)** guanine metabolism and **(C)** adenine ribonucleotide degradation. Reduction from control is indicated by a downward red arrow. Downstream and upstream metabolites according to the KEGG pathway for purine metabolism are indicated. The box and whiskers summarize the normalized values with mean indicated by a yellow diamond and the central line indicating the median black dots representing the values from all samples (*n* = 3). Box and whiskers were generated using MetaboAnalyst 6.0 and edited for visual clarity in Inkscape. Figure contains diagrams contextualizing the role of the metabolites in purine biosynthesis and adenine ribonucleotide degradation. Chemical structures were created using ChemDraw. ****p < 0.0001.

Numerous metabolites involved in purine metabolism were identified as altered (22/70) following 0.5 M (NH_4_)_2_SO_4_ cultivation (Figure 5A). Figure 6 depicts boxplots of the metabolites within purine metabolism that were found to be significantly altered in both the pathway and volcano analysis—glutamine (*p* < 0.0001), deoxyguanosine (*p* < 0.001), guanosine (*p* < 0.0001), hypoxanthine (*p* < 0.0001) and inosine (*p* < 0.0001). The KEGG pathway for purine metabolism attributes these molecules to purine biosynthesis (glutamine) (Figure 6A), guanine metabolism (deoxyguanosine, guanosine) (Figure 6B) and adenine ribonucleotide degradation (hypoxanthine, inosine) (Figure 6C). Pathway analysis also identified 3′,5′-cyclic-GMP as significantly elevated (*p* < 0.05) and adenine as significantly reduced (*p* < 0.05) in 0.5 M (NH_4_)_2_SO_4_ cultivated samples compared to control, further suggesting reduced turnover of 3′,5′-cyclic-GMP for production of guanine and reduced adenine biosynthesis.

### Changes to amino acids detected in (NH_4_)_2_SO_4_ cultivated Slthf1

**Figure 7:**
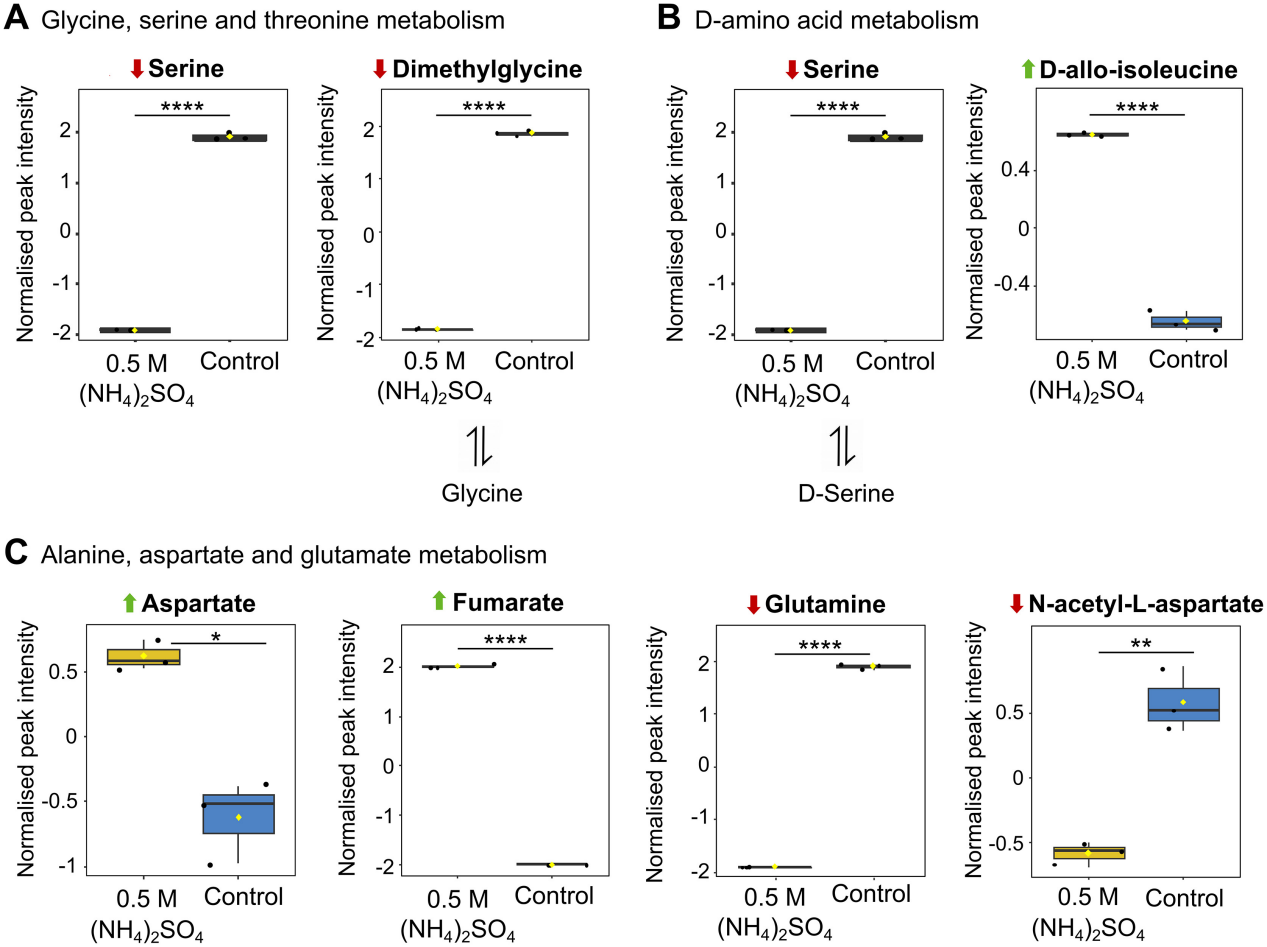
Altered metabolites of amino acid metabolism. Box plots showing significantly altered metabolites involved in **(A)** glycine, serine and threonine metabolism **(B)** D-amino acid metabolism and **(C)** alanine, aspartate and glutamate metabolism. Reduction and elevation from control is indicated by a downward red arrow and upward green arrow, respectively. Downstream and upstream metabolites according to the relevant KEGG pathway are indicated. The box and whiskers summarize the normalized values with mean indicated by a yellow diamond and the central line indicating the median black dots representing the values from all samples (*n* = 3). Box and whiskers were generated using MetaboAnalyst 6.0 and edited for visual clarity in Inkscape. *p < 0.05; **p < 0.01; ****p < 0.0001.

Pathway analysis indicated four amino acid pathways altered in response to 0.5 M (NH_4_)_2_SO_4_ cultivation (Figure 5A). Box plots of the altered metabolites identified in the pathway analysis related to amino acid metabolism that were also found to be altered in the volcano analysis are depicted in Figure 7. These correspond to the metabolism of glycine, serine and threonine (*p* < 0.001) (Figure 7A), D-amino acids (*p* < 0.01) (Figure 7B) and alanine, aspartate and glutamate (*p* < 0.001) (Figure 7C). All comparisons made below reference metabolites altered in samples cultivated in 0.5 M (NH_4_)_2_SO_4_ compared to control samples. Five metabolites out of 33 total metabolites were found to be altered in glycine, serine and threonine metabolism. Of these, two were found to be significantly reduced: serine (*p* < 0.0001) and dimethylglycine (*p* < 0.0001). Reduction of serine may account for the reduction to PS. Four metabolites related to D-amino acid biosynthesis were found to be altered. These included significantly lower levels of serine (*p* < 0.0001). It is notable that D-allo-isoleucine was also found to be significantly elevated in the volcano analysis (*p* < 0.001). In alanine, aspartate and glutamate metabolism, 11/28 metabolites were found to be altered. These included significantly higher levels of aspartate (*p* < 0.01), fumarate (*p* < 0.0001), and significantly lower levels of N-acetyl-L-aspartate (*p* < 0.01), glutamate (*p* < 0.05), glutamine (*p* < 0.0001) and succinate (*p* < 0.05). Not depicted in Figure 7, the significant reduction to the levels of glutamate (*p* < 0.05) and glutamine (*p* < 0.0001), and significant elevation to the levels of aspartate (*p* < 0.01) and fumarate (*p* < 0.0001), were also relevant to arginine metabolism.

### Differential metabolite abundance indicates modulations to energy and carbon metabolism

**Figure 8:**
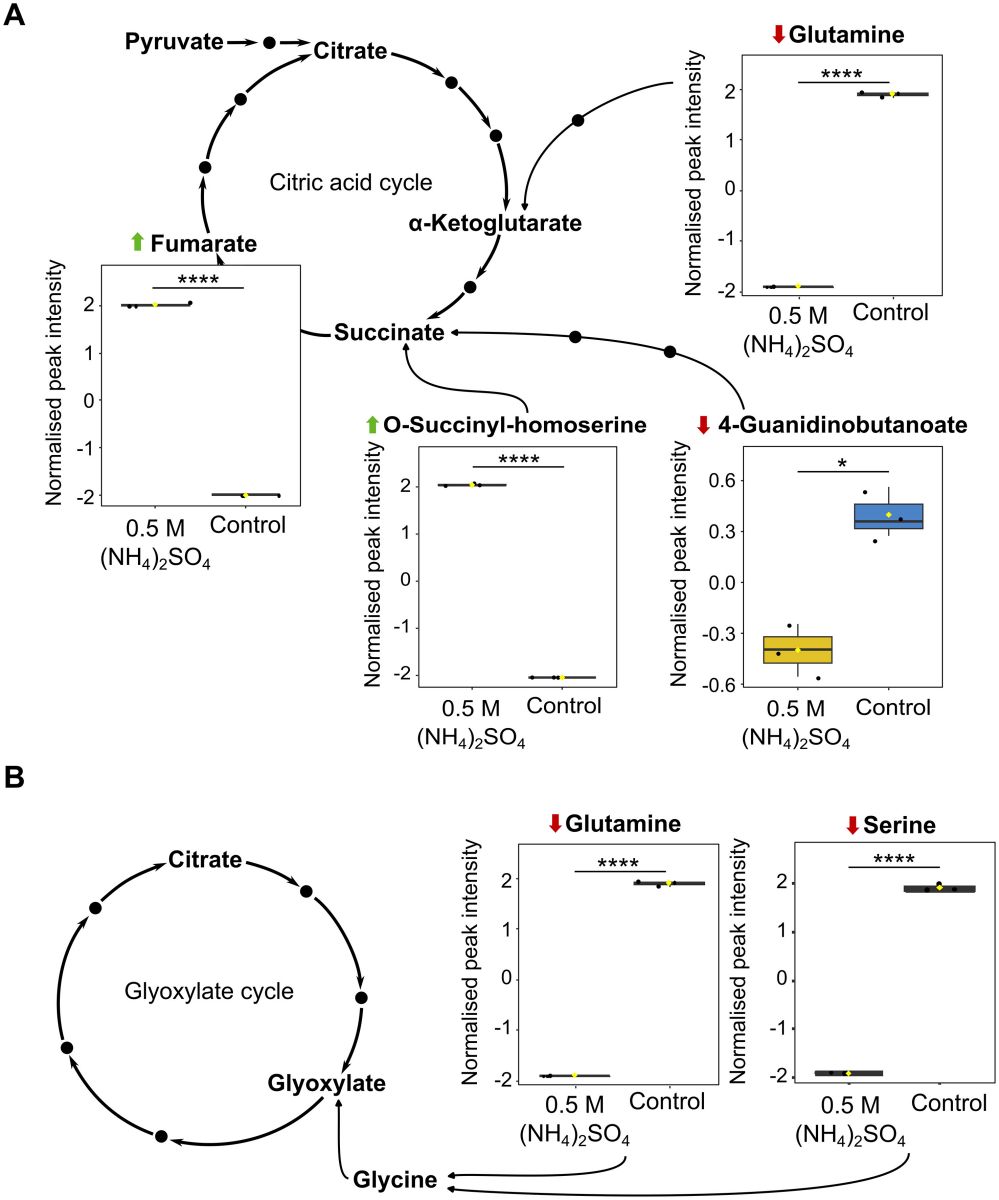
Recognized metabolites of carbon and energy metabolism. Box plots showing significantly altered metabolites involved in **(A)** the citric acid cycle and **(B)** the glyoxylate cycle. Reduction and elevation from control is indicated by a downward red arrow and upward green arrow, respectively. Downstream and upstream metabolites according to the relevant KEGG pathway are depicted or indicated by a solid black dot. The box and whiskers summarize the normalized values with mean indicated by a yellow diamond and the central line indicating the median black dots representing the values from all samples (*n* = 3). Box and whiskers were generated using MetaboAnalyst 6.0 and edited for visual clarity in Inkscape. ns, no significance; *p < 0.05; ****p < 0.0001.

The citric acid cycle was found to be significantly altered (*p* < 0.05) in the pathway analysis (Figure 5A). Pathway analysis revealed significant reduction to succinate (*p* < 0.05) and elevation to fumarate (*p* < 0.0001) in the 0.5 M (NH_4_)_2_SO_4_ cultivated sample compared to control. It is notable that elevated levels of O-succinyl-homoserine (FC = 1.44 × 10^9^, *p* < 0.0001), a succinate precursor, and reduced levels of 4-Guanidinobutanoate (FC = 0.431, *p* < 0.05), a product of arginine degradation which is subsequently converted to succinate, were also identified in the volcano analysis. Figure 8A depicts box plots of significantly altered metabolites in the citric acid cycle identified in both the pathway and volcano analysis. Pyruvate metabolism was also found to be significantly altered (*p* < 0.01) with significant alteration to fumarate (*p* < 0.0001), and non-significant alteration of (S)-lactate (*p* = 0.269) and pyruvate (*p* = 0.116). Reduced levels of glutamate (*p* < 0.05), succinate (*p* < 0.05), and acetoacetate (*p* = 0.119) in the 0.5 M (NH_4_)_2_SO_4_ cultivated cells compared to control also caused butanoate metabolism to be found as significantly altered (*p* < 0.01). The most significantly altered pathway was that of glyoxylate and dicarboxylate metabolism (*p* < 0.001). Three metabolites of this pathway were found to be significantly reduced in the 0.5 M (NH_4_)_2_SO_4_ cultivated cells compared to control: glutamate (*p* < 0.05), glutamine (*p* < 0.0001) and serine (*p* < 0.0001). Figure 8B depicts box plots of significantly altered metabolites in the glyoxylate cycle identified in both the pathway and volcano analysis. Notably, we additionally found the methylaspartate cycle intermediate N-methylaspartate to be reduced in the volcano analysis (FC = 0.108, *p* < 0.05) in (NH_4_)_2_SO_4_ cultivated cells (Figure 3B).

## Discussion

To assess the habitability of environments on other celestial bodies, it is valuable to first establish the known limits of life on Earth. (NH_4_)_2_SO_4_ could be a constituent of the surface of Europa (Mermy et al., 2023) delivered from the ocean below, and a major salt within the subsurface ocean of Titan (Fortes et al., 2007; Grindrod et al., 2008). These icy moons have strong astrobiological interest due to the presence of liquid water (Carr et al., 1998; Pappalardo et al., 1998; Bills and Nimmo, 2011; Nimmo and Pappalardo, 2016) and putative physicochemical properties suitable for the emergence of life. In this work, we provide insights into the relationship between (NH_4_)_2_SO_4_ and environmental habitability by investigating molar thresholds for growth, alterations to morphology and the metabolite profile of a hydrothermal vent extremophile, Slthf1, cultivated in (NH_4_)_2_SO_4_.

Our work showed that concentrations at and exceeding 0.25 M (NH_4_)_2_SO_4_ caused a distinct alteration to growth rate, while concentrations at and exceeding 0.75 M reduced final cell density. The molar limits established agree with that determined in *B. subtilis*, in which optical density when cultivated in (NH_4_)_2_SO_4_ remained unchanged until 0.76 M (Hamill et al., 2020). However, reduction to growth rate has been recorded at a higher molar limit in *B. subtilis*−0.375 M (Müller et al., 2006) and 0.5 M (NH_4_)_2_SO_4_ (Hamill et al., 2020), as well as in *Escherichia coli* (0.375 M (NH_4_)_2_SO_4_), and *C. glutamicum* (1 M (NH_4_)_2_SO_4_) (Müller et al., 2006). Slthf1 did not exhibit complete cell death at 1 M (NH_4_)_2_SO_4_, as also observed for *C. glutamicum* at this concentration (Müller et al., 2006). Thus, we propose concentrations up to 1 M (NH_4_)_2_SO_4_ do not limit habitability for Slthf1 but do affect cell density and growth rate.

In agriculture, application of (NH_4_)_2_SO_4_ fertilizer is found to alter bacterial community structure and diversity; these effects are attributed to intrinsic changes in pH (Khonje et al., 1989; Toljander et al., 2008; Zhang et al., 2017). Our results show pH-independent influence of (NH_4_)_2_SO_4_ on bacterial density. In *B. subtilis, E. coli*, and *C. glutamicum*, changes to growth kinetics upon treatment with (NH_4_)_2_SO_4_ or Na_2_SO_4_ have been found to be near identical, indicating toxicity by osmolarity or ionic strength as opposed to the specific effects of (NH_4_)_2_SO_4_ (Müller et al., 2006). We advance the current understanding of the limits of life in (NH_4_)_2_SO_4_ by presenting a new perspective on ammonium salt toxicity in Slthf1 – we indicate the presence of both ${\text{NH}}_{4}^{+}$ and ${\text{SO}}_{4}^{2-}$ ions, as opposed to individual ionic toxicity, salinity, osmotic or ionic strength, as the source of reduction to growth rate and cell density. Our experiments show that when ionic concentrations of ${\text{NH}}_{4}^{+}$ were equal to 1 M (0.5 M (NH_4_)_2_SO_4_), there was no statistical difference between growth in 0.5 M (NH_4_)_2_SO_4_ and 1 M NH_4_Cl, but growth was comparatively reduced in 1 M NH_4_NO_3_. Likewise, when ${\text{SO}}_{4}^{2-}$ concentrations were equal to 1 M (1 M (NH_4_)_2_SO_4_, 2 M ${\text{NH}}_{4}^{+}$) the levels of cell density were significantly less compared to those in 1 M Na_2_SO_4_ brines also containing 1 M SO_4_ (2 M Na^+^). There was no correlation between NH_4_ and ${\text{SO}}_{4}^{2-}$ toxicity separately, and changes in cell density were also not accounted for by differences in pH, water activity, osmolarity, ionic strength or salinity.

(NH_4_)_2_SO_4_ is the most kosmotropic salt utilized in this study, while NH_4_NO_3_ and Na_2_SO_4_ represent the most chaotropic salts (Cacace et al., 1997; Zhang and Cremer, 2006). However, chaotropic and kosmotropic properties alone are not typically predictors of habitability (Stevens and Cockell, 2020). We propose (NH_4_)_2_SO_4_ may repress cell density due to altered ${\text{NH}}_{4}^{+}$ assimilation promoted by the metabolism of ${\text{SO}}_{4}^{2-}$, the effects of which become apparent when concentrations of ${\text{NH}}_{4}^{+}$ exceed 1 M. Indeed, untargeted metabolomics revealed O-succinyl-homoserine was found to be significantly elevated in (NH_4_)_2_SO_4_ cultivated Slthf1. This metabolite is part of the sulfur assimilation pathway (Vermeij and Kertesz, 1999; Ferla and Patrick, 2014; Kim et al., 2024).

Significantly lowered levels of glutamine, a nitrogen assimilation metabolite, were also detected in Slthf1 cultivated in (NH_4_)_2_SO_4_. This reduction coincides with other studies of ${\text{NH}}_{4}^{+}$ stress, whereby genes in nitrogen assimilation have been found to be altered in *Nitrobacter winogradskyi* (Sayavedra-Soto et al., 2015), and nitrogen reduction reactions reduced in *Methylomonas sp*. ZR1 (Guo W. et al., 2024). Under low ${\text{NH}}_{4}^{+}$ conditions nitrogen metabolism occurs by the glutamine synthetase-glutamate synthase (GS-GOGAT) pathway, whereby ${\text{NH}}_{4}^{+}$ is converted to glutamine and subsequently to glutamate, the primary nitrogen reservoir (Nagatani et al., 1971; Bravo and Mora, 1988; Schreier et al., 1993). Under high ${\text{NH}}_{4}^{+}$ conditions, the glutamate dehydrogenase (GDH) pathway predominates, whereby α-ketoglutarate and ${\text{NH}}_{4}^{+}$ are converted to glutamate (Kanamori et al., 1987; Reitzer, 2003; Legendre et al., 2020). We propose Slthf1 utilized the GDH pathway under high (NH_4_)_2_SO_4_. The GDH pathway produces glutamate, but does not rely on glutamine, which coincides with the *t*-test results showing non-significant change in glutamate and significant reduction to glutamine in (NH_4_)_2_SO_4_ cultivated Slthf1 compared to the control sample without (NH_4_)_2_SO_4_. Similarly, a downregulation to transcripts in the GS-GOGAT pathway has been observed in *Enterobacter cloacae* HNR under NH_4_^+^ stress (Weng et al., 2022), but conversely upregulated in *Nitrobacter winogradsky* Nb-255 (Sayavedra-Soto et al., 2015). Due to a lower affinity for NH_4_^+^, GDH is less efficient in producing glutamate than GS (Wakisaka et al., 1989; Yan et al., 1996), which may contribute to the observed reduction in growth rate of (NH_4_)_2_SO_4_ cultivated Slthf1. The underlying cause for GDH-dependent synthesis of glutamate is beyond the scope of this study but can be speculated. GS may be regulated by the Na^+^/K^+^ pump or Ca^2+^ (Benjamin, 1987), and is linked to an intracellular K^+^ pool (Yan et al., 1996). Given that ${\text{NH}}_{4}^{+}$ can compete with K^+^ transport through ion channels (Moser, 1987) and has also been implicated in disrupting Ca^2+^ homeostasis (Wang et al., 2018), it is possible the presence of ${\text{NH}}_{4}^{+}$ could disrupt the internal K^+^ and Ca^2+^ balance that regulates GS activity. Notably, no GS or GOGAT activity has been detected in *B. pasteurii* grown in 0.04 M ${\text{NH}}_{4}^{+}$ (Mörsdorf and Kaltwasser, 1989), an organism that has shown ammonia-dependent oxidation of glutamate (Wiley and Stokes, 1962, 1963).

Transamination reactions with glutamate generate amino acids, purines and pyrimidines, and catabolism of glutamate provides intermediates for the citric acid cycle (Commichau et al., 2006; Walker and van der Donk, 2016). We observed reduced levels of amino acids (serine and N-acetyl-L-aspartate) and reduced metabolites in guanine synthesis, adenine synthesis and adenine degradation (guanosine, inosine, hypoxanthine) in Slthf1 under (NH_4_)_2_SO_4_ cultivation. These changes could indicate: (i) implementation of energy saving adaptations such as reducing amino acid and nucleotide biosynthesis, (ii) a shift to catabolism for energy production as suggested by an inferred reduction to amino acid pools and nucleotides, and (iii) increased turnover of guanosine, inosine and hypoxanthine for synthesis of energy carrier molecules guanosine triphosphate (GTP) and adenosine triphosphate (ATP).

Lower levels of amino acids and purine metabolites have also been identified in *Pseudomonas* RCH2 when grown in media without ammonia (Kurczy et al., 2016). Nutrient-limiting conditions promote catabolic processes. Under stress, cells require more energy to sustain protective and adaptive responses. It is therefore plausible changes to guanosine, inosine, hypoxanthine levels were reflective of an internal stress response to meet energy demands in response to (NH_4_)_2_SO_4_. In accordance with this, we observe the relative abundance of citric acid cycle intermediate fumarate to be elevated in (NH_4_)_2_SO_4_ cultivated cells. However, we also observed reduced levels of citric acid cycle intermediate succinate. In the canonical citric acid cycle, succinate is oxidized to fumarate by succinate dehydrogenase. The differential alterations to these metabolites could indicate upregulation of the succinate to fumarate conversion, or reduced input from the glyoxylate cycle under stress conditions. In the latter case, succinate is liberated by cleavage of isocitrate and funneled into the citric acid cycle. A reduction to metabolites in the glyoxylate cycle in Slthf1 was indicated by pathway analysis. We also found several precursor molecules to key intermediates in the citric acid cycle and butanoate metabolism were reduced. This is suggestive of altered carbon metabolism. Indeed, citric acid cycle genes have been found to be down regulated in *E. cloacae* HNR exposed to high ${\text{NH}}_{4}^{+}$ (Weng et al., 2022).

The reduced levels of metabolites in citric acid cycle, the glyoxylate cycle and butanoate cycle suggest utilization of an alternative mechanism of catabolism. Notably, fumarate also feeds the methylaspartate cycle. We find the methylaspartate cycle intermediate N-methylaspartate to be reduced in (NH_4_)_2_SO_4_ cultivated cells. The methylaspartate cycle has been characterized in haloarchaea and involves the processing of acetyl-CoA by a series of reactions to malate, a starting substrate for anabolism. Within this process, methylaspartate is converted to N-methylaspartate and then mesaconate (Khomyakova et al., 2011; Borjian et al., 2016). Albeit reduced, the detection of N-methylaspartate could indicate an active methylaspartate cycle, in turn indicating a less active glyoxylate cycle. We can surmise these alterations to key intermediates and precursor molecules as an indication of altered energy and carbon metabolism induced by high concentrations of (NH_4_)_2_SO_4_. This aligns with previous studies that show ${\text{NH}}_{4}^{+}$ exposed bacteria alter central carbon pathways and the citric acid cycle (Sugden et al., 2021; Guo L. et al., 2024).

Currently, the planetary habitability and compositions of extraterrestrial aqueous environments, until measured, can only be speculated. The ocean of Europa is estimated to be predominantly composed of MgSO_4_ (McCord et al., 1998; Kargel et al., 2000; Zolotov and Shock, 2001) or chloride salts (Brown and Hand, 2013; Hand and Carlson, 2015; Ligier et al., 2016), and thus could have a lower concentration of ammonia and (NH_4_)_2_SO_4_, if any, than Titan (Kargel, 1991). Titan is expected to have formed with up to 15% ammonia (Lunine and Stevenson, 1987; Engel et al., 1994; Tobie et al., 2005) and could contain an ocean of (NH_4_)_2_SO_4_ (Fortes et al., 2007; Grindrod et al., 2008). Our results showed a reduction to growth rate and cell density with increasing (NH_4_)_2_SO_4_. However, we found that Slthf1 cells remained viable at concentrations up to 1 M (NH_4_)_2_SO_4_ (2 M ${\text{NH}}_{4}^{+}$). This data cannot suggest whether icy moons oceans are or have been inhabited but can suggest that substantial concentrations of (NH_4_)_2_SO_4_ may not necessarily preclude survival of terrestrial bacteria in highly concentrated (NH_4_)_2_SO_4_ aqueous environments. Such conditions could be relevant to the subsurface oceans hypothesized on icy moons like Europa and Titan. The findings reported in this study also have terrestrial applications. For instance, application of 35 g/m^2^ of (NH_4_)_2_SO_4_ fertilizer, as advised for some commercial fertilizers, could yield a molarity of 2.64 M when dissolved in 100 mL water. At concentrations equal to and below 1 M, our results showed that (NH_4_)_2_SO_4_ slowed growth rate and reduced cell density, as well as altered metabolites associated with growth processes in nitrogen, carbon and energy metabolism, purine metabolism and amino acid metabolism. These cellular and molecular effects could correlate with alterations to bacterial populations, richness and diversity observed in literature when soil is treated with (NH_4_)_2_SO_4_ (Gorissen et al., 1993; Witter et al., 1993; Toljander et al., 2008). This further establishes that (NH_4_)_2_SO_4_ can affect susceptible terrestrial bacteria when applied.

Stress responses and metabolites in bacteria can act as potential biomarkers for life (Kort et al., 2008; Goordial et al., 2017; Moreno-Paz et al., 2023). For this reason, instruments capable of metabolite detection have been considered for life-detection missions (Weber et al., 2023; Wronkiewicz et al., 2024). Under (NH_4_)_2_SO_4_ cultivation, we detected higher levels of phospholipids PC and PE with monounsaturated 16:1 and 18:1 lipids in Slthf1 cultivated in (NH_4_)_2_SO_4_. Similar biomarkers have been reported in heterotrophic nitrification-aerobic denitrification (HN-AD) bacteria and *E. cloacae* HNR exposed to high ${\text{NH}}_{4}^{+}$ (Weng et al., 2022; Guo L. et al., 2024). In halophiles, this modification may support survival under salt stress by enhancing membrane fluidity (Lopalco et al., 2013). Fatty acids are also synthesized by halophiles under salt stress (Liu et al., 2015); we observed a small elevation to stearic acid in Slthf1. However, given the low salinity of the (NH_4_)_2_SO_4_ solution, Sltfh1 did not demonstrate many other metabolomic markers characteristic of osmotic stress (e.g., accumulation of compatible solutes) (Saum and Müller, 2008). We recognize the (NH_4_)_2_SO_4_ media utilized in this study was simplistic. This was intentional, as we aimed to probe the specific effect of (NH_4_)_2_SO_4_ on life. A natural progression of this work would be to investigate survival limits and physiology under multi-extremes. Brines simulating the putative composition of fluids in the oceans of Europa and Titan (such as the incorporation of MgSO_4_ or sodium ions) would be particularly valuable in identifying physiological markers of life in aqueous (NH_4_)_2_SO_4_ environments.

## Data Availability

The original contributions presented in the study are included in the article/Supplementary material, further inquiries can be directed to the corresponding author.
