# Supplementary material for: Growth, physiology, and metabolism of Halomonas meridiana in aqueous ammonium sulfate with implications for icy moon astrobiology
Source: Front Microbiol. 2025 Sep 19;16:1642998. doi: 10.3389/fmicb.2025.1642998 (PMC12492958; doi:10.3389/fmicb.2025.1642998)
Supplement: Supplementary file 1 [file Data_Sheet_1.pdf]

Supplementary Material

Frontiers in Microbiology

Growth, physiology and metabolism of *Halomonas meridiana* in aqueous ammonium sulfate with implications for icy moon astrobiology

Cassie M. Hopton[1*], Peter Nienow[2], Charles S. Cockell[1],

[1] UK Centre for Astrobiology, School of Physics and Astronomy, University of Edinburgh, United Kingdom

[2] School of Geosciences, University of Edinburgh, United Kingdom

*Corresponding author, c.m.hopton@sms.ed.ac.uk,

Supplementary Figure S1. Cell viability and optical density at 600 nm over 24 h. Column graph shows the PrestoBlue™ cell viability (black bars) against optical density at 600 nm (OD_{600}) (striped bars) of *Halomonas meridiana* sampled at 0, 2, 4, 6 and 24 hours. Column heights represent the mean ± s.d. (n = 3).

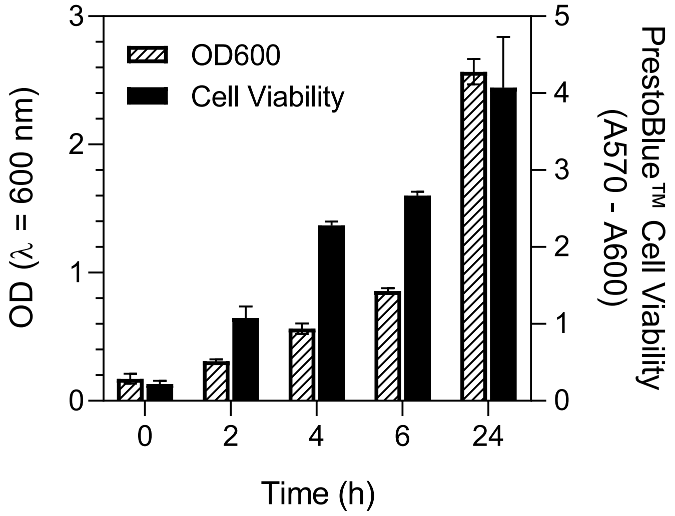

Supplementary Table S1. Statistical *p*-values for the mean OD_{600} of *H. meridiana* after 48 h growth in 0.5 M $(NH_4)_2SO_4$ vs. 0.5 M sulfate salts and 0.25 M $(NH_4)_2SO_4$ vs. 0.5 M ammonium salts, including HCl pH-matched solutions

	0.5 M					
	NH_4Cl	NH_4NO_3	HCl pH 5.9	Na_2SO_4	K_2SO_4	HCl pH 5.8
0.25 M $(NH_4)_2SO_4$	0.359	0.765	0.636			
0.5 M $(NH_4)_2SO_4$				>0.999	>0.999	>0.999

Supplementary Table S2. Statistical *p*-values for the mean a_w of $(NH_4)_2SO_4$ vs. sulfate salt and ammonium salt solutions at concentrations of 0.1 M, 0.5 M and 1 M.

	0.1 M			
	NH_4Cl	NH_4NO_3	Na_2SO_4	K_2SO_4
0.05 M $(NH_4)_2SO_4$	0.0558	0.233		
0.1 M $(NH_4)_2SO_4$			0.205	0.133
	0.5 M			
	NH_4Cl	NH_4NO_3	Na_2SO_4	K_2SO_4
0.25 M $(NH_4)_2SO_4$	>0.999	>0.999		
0.5 M $(NH_4)_2SO_4$			0.382	0.448
	1 M			
	NH_4Cl	NH_4NO_3	Na_2SO_4	
0.5 M $(NH_4)_2SO_4$	0.624	0.999		
1 M $(NH_4)_2SO_4$			0.126	

Supplementary Table S3. Metabolites significantly altered (*p*-value lower than 0.05, FDR corrected) between the treatment conditions assessed by unpaired *t*-test (*n* = 3). Unpaired t-test analysis was performed using the web-based software MetabAnalyst 6.0.

Metabolite	t.stat	p.value	-log10(p)	FDR
O-Succinyl-homoserine	284.81	9.12E-10	9.0401	0.000000482
Lumazine	-215.6	2.78E-09	8.5565	0.000000734
Fumarate	157.57	9.73E-09	8.0118	0.00000172
Poly-(glycine)5	118.74	3.02E-08	7.5204	0.00000281
N,N-Dimethylglycine	-115.03	3.42E-08	7.4654	0.00000281
N6-(delta2-Isopentenyl)-adenine	110.46	4.03E-08	7.3949	0.00000281
Glutamine	-109.44	4.18E-08	7.3787	0.00000281
4-Coumarate	-108.97	4.25E-08	7.3713	0.00000281
Dihydrobiopterin	87.054	0.000000104	6.9814	0.00000558
L-Serine	-86.816	0.000000106	6.9766	0.00000558
NVNDVIAPAFVK (Tryptic Peptide)	-80.982	0.000000139	6.8558	0.00000665
Hypoxanthine	-79.387	0.000000151	6.8213	0.00000665
Glutarylcarnitine	-75.613	0.000000183	6.7367	0.00000746
2-Deoxyadenosine-5-monophosphate	53.753	0.000000717	6.1444	0.0000271
PC 36:05	48.724	0.00000106	5.974	0.0000365
Indole-3-ethanol	-48.248	0.0000011	5.957	0.0000365
Inosine	-38.925	0.0000026	5.5847	0.000081
Imazapyr	-37.711	0.00000295	5.5297	0.0000828
Pyridoxine	-37.641	0.00000297	5.5265	0.0000828
PE 37:01	34.213	0.00000435	5.3611	0.00011517
D-allo-Isoleucine	31.901	0.00000576	5.2399	0.00014499
Indole-3-pyruvate	-31.113	0.00000636	5.1966	0.0001529
Guanosine	-28.125	0.00000951	5.0219	0.0002187
PS 40:01	-23.28	0.0000202	4.6951	0.00044474
Deoxyguanosine	-22.083	0.0000249	4.604	0.00052664
2-Amino-2-methylpropanoate	-20.833	0.0000314	4.5035	0.0006383
Stearic Acid (18:0)	16.285	0.0000832	4.0798	0.0016302
2-Methylnaphthalene	15.07	0.000113	3.9469	0.0021349
Oxoproline	-13.984	0.0001517	3.819	0.0027672
Protoporphyrin	-13.423	0.00017817	3.7492	0.0031417

Supplementary Table S4. Volcano analysis comparing molecular features in the 0.5 M $(NH_4)_2SO_4$ dataset against the control dataset (*n* = 3), where metabolites identified in the analysis exhibited a fold change (FC) greater than 2 and a *p*-value < 0.05 (adjusted using FDR correction). Volcano analysis was performed using the web-based software MetabAnalyst 6.0.

Metabolite	FC	log2(FC)	p.ajusted	-log10(p)
Glutarylcarnitine	1.23E-09	-29.595	7.46E-06	5.1272
Hypoxanthine	1.47E-09	-29.345	6.65E-06	5.177
Deoxyguanosine	1.50E-09	-29.31	0.00052664	3.2785
Indole-3-ethanol	1.72E-09	-29.115	3.65E-05	4.4377
Guanosine	3.16E-09	-28.239	0.0002187	3.6601
NVNDVIAPAFVK (Tryptic Peptide)	3.55E-09	-28.07	6.65E-06	5.177
Inosine	5.10E-09	-27.547	8.10E-05	4.0917
4-Coumarate	6.19E-09	-27.266	2.81E-06	5.5509
Lumazine	8.84E-09	-26.753	7.34E-07	6.1341
Glutamine	1.15E-08	-26.371	2.81E-06	5.5509
L-Serine	1.20E-08	-26.312	5.58E-06	5.2532
N,N-Dimethylglycine	2.81E-08	-25.083	2.81E-06	5.5509
Protoporphyrin	3.11E-08	-24.94	0.0031417	2.5028
Imazapyr	3.46E-08	-24.784	8.28E-05	4.0818
Indole-3-pyruvate	4.16E-08	-24.517	0.0001529	3.8156
Pyridoxine	5.48E-08	-24.12	8.28E-05	4.0818
PS 40:01	3.16E-07	-21.592	0.00044474	3.3519
N-Methylaspartate	0.10785	-3.2129	0.034078	1.4675
Oxoproline	0.11152	-3.1646	0.0027672	2.558
2-Amino-2-methylpropanoate	0.13412	-2.8984	0.0006383	3.195
2-Acetamindo-2-deoxy-beta-D-glycosylamine	0.1417	-2.8191	0.010995	1.9588
N-acetyl-L-aspartate	0.15917	-2.6514	0.026225	1.5813
Nicotinamide	0.30151	-1.7297	0.041236	1.3847
4-Guanidinobutanoate	0.43137	-1.213	0.03852	1.4143
Trigonelline	2.2167	1.1484	0.032461	1.4886
Stearic Acid (18:0)	2.4299	1.2809	0.0016302	2.7877
PC (18:1/18:1) (del9-trans)	4.8262	2.2709	0.0074116	2.1301
4-Quinolinecarboxylate	7.1661	2.8412	0.026225	1.5813
L-aspartate	7.2425	2.8565	0.03852	1.4143
D-allo-Isoleucine	8.4184	3.0735	0.00014499	3.8387
PC(16:1(9Z)/16:1(9Z))	11.35	3.5046	0.026225	1.5813
PE (O-34:03)	46.154	5.5284	0.022706	1.6439
2-Deoxyadenosine-5-monophosphate	5141100	22.294	2.71E-05	4.5671
2-Methylnaphthalene	41599000	25.31	0.0021349	2.6706
PE 37:01	70589000	26.073	0.00011517	3.9387

Poly-(glycine)5	95621000	26.511	2.81E-06	5.5509
N6-(delta2-Isopentenyl)-adenine	122200000	26.865	2.81E-06	5.5509
Dihydrobiopterin	200910000	27.582	5.58E-06	5.2532
Fumarate	743070000	29.469	1.72E-06	5.7655
O-Succinyl-homoserine	1.44E+09	30.421	4.82E-07	6.3166
PC 36:05	1559700000	30.539	3.65E-05	4.4377

Supplementary Table S5. Outcome of the pathway analysis depicting pathways identified in the metabolomics dataset as significantly changed (*p*-value = < 0.05; FDR = < 0.05). Pathway analysis was performed using the web-based software MetabAnalyst 6.0.

Pathway	Match	*p*-value	FDR
Sphingolipid metabolism	1/3	1.05×10^{-7}	6.54×10^{-6}
Nitrogen metabolism	2/6	5.71×10^{-7}	1.77×10^{-5}
Purine metabolism	22/70	5.36×10^{-5}	0.00111
Glyoxylate and dicarboxylic acid metabolism	7/32	3.03×10^{-4}	0.00382
Glycine, serine and threonine metabolism	5/33	3.08×10^{-4}	0.00382
Arginine biosynthesis	7/14	4.43×10^{-4}	0.00414
Alanine, aspartate and glutamate metabolism	11/28	4.67×10^{-4}	0.00414
D-amino acid metabolism	4/15	0.00352	0.0249
Folate biosynthesis	2/26	0.00361	0.0249
Pyruvate metabolism	3/23	0.00405	0.0251
Butanoate metabolism	3/15	0.00454	0.0256
TCA cycle	5/20	0.00862	0.0445
